# Timing and Reported Indications for Revision After Anatomic Versus Reverse Total Shoulder Replacement: A National Joint Registry Study

**DOI:** 10.3390/jcm15218381

**Published:** 2026-10-28

**Authors:** Sedeek Mosaid, Banah Khoshnaw, Nuala Pepper, Omar Salem, Paul Lee, Charles Corbin

**Affiliations:** 1 Department of Trauma and Orthopaedic Surgery, United Lincolnshire Teaching Hospitals NHS Trust, Lincoln LN2 5QY, UK; 2 Chelsea and Westminster Hospital NHS Foundation Trust, London SW10 9NH, UK

**Keywords:** shoulder arthroplasty, reverse total shoulder replacement, anatomic total shoulder replacement, revision, glenohumeral osteoarthritis, rotator cuff tear arthropathy, National Joint Registry, implant survivorship

## Abstract

**Background/Objectives:** Although reverse total shoulder replacement (RTSR) is now the predominant shoulder replacement procedure in England and Wales, RTSR and anatomic total shoulder replacement (anatomic TSR) serve mainly different indications and are seldom compared in terms of when and why they are revised. We aimed to describe the timing and reported indications for the first revision of each construct and quantify the between-construct comparison that the National Joint Registry (NJR) describes only qualitatively. **Methods:** We conducted a secondary analysis of published aggregate data from the NJR’s 22nd Annual Report for elective primary anatomic TSR (*n* = 21,180) and RTSR (*n* = 39,408) performed in England and Wales between 2012 and 2024. We used NJR’s published Kaplan–Meier and prosthesis-time incidence rate (PTIR) estimates to derive the unadjusted between-construct difference (anatomic TSR minus RTSR) in cumulative revision at each time point, with approximate 95% confidence intervals (CIs), and rate ratios for each reported revision indication. **Results:** In these unadjusted estimates, the between-construct difference was −0.66 percentage points at 1 year (95% CI [−0.85, −0.47]), close to 0 at 3 years, and 3.52 percentage points at 12 years (95% CI [2.19, 4.85]). Revision for infection (rate ratio, 2.43), periprosthetic fracture (1.75) and instability (1.26) was more common after RTSR, whereas cuff insufficiency was markedly less frequent (PTIR 0.01 vs. 0.34 per 100 prosthesis years). **Conclusions:** The constructs differ in revision timing and reported indications. Because they are used for different indications in patients of different ages, these unadjusted data are descriptive and cannot establish comparative superiority or guide construct selection alone.

## 1. Introduction

Shoulder replacement reliably relieves pain in end-stage glenohumeral disease, and most constructs survive well into the second decade. A systematic review and meta-analysis of series and registry reports with more than 10 years of follow-up estimated that approximately 90% remain unrevised at 10 years [1]. In the United States, the number of individuals living with a shoulder construct and therefore at risk of revision is rising steeply, with a projected increase in the burden of revision surgery [2,3].

Practice has changed faster than the comparative evidence supporting it. Conceived by Grammont and Baulot for a cuff-deficient shoulder [4], reverse total shoulder replacement (RTSR) medialises and distalises the glenohumeral centre of rotation and substitutes deltoid function for a deficient rotator cuff; this biomechanical reconfiguration underlies both its success in cuff-deficient shoulders and its characteristic complications [5,6]. Once confined to cuff tear arthropathy, RTSR has expanded rapidly internationally into a widening range of indications [7]. A meta-analysis of 11 public joint registries found that RTSR use had almost doubled, become near-universal for cuff tear arthropathy, and reached near-parity with anatomic total shoulder replacement (anatomic TSR) for osteoarthritis, despite limited high-quality comparative evidence for indications beyond cuff tear arthropathy [7]. An earlier international registry comparison showed wide variation between countries in the type of procedure chosen within major indications [8].

Studies using the National Joint Registry (NJR) and Hospital Episode Statistics (HES) for England have addressed specific comparative questions: RTSR versus anatomic TSR for osteoarthritis with an intact cuff [9], hemiarthroplasty versus anatomic TSR for osteoarthritis [10], the lifetime risk of reoperation and serious adverse events [11] and the association between surgeon volume and revision [12]. What is less clearly articulated in the general orthopaedic literature is how the timing and the reported indications for revision of the two dominant constructs compare across a whole NJR cohort: whether they differ not merely in how frequently they are revised, but in when revision occurs and the indication recorded for it.

However, comparing the two constructs requires careful framing because they are largely used for different indications. RTSR was developed for the cuff-deficient shoulder and is the construct used for almost all cuff tear arthropathy, typically in older patients, whereas anatomic TSR is primarily used for primary osteoarthritis with an intact rotator cuff, typically in younger patients [13]. The two constructs directly compete principally in the subgroup with osteoarthritis and an intact cuff, and even there, the choice remains debated and has been identified as a national research priority [9,14]. Therefore, any registry comparison of the constructs as a whole compares not only two constructs but also two different indications and two different patient populations, and unadjusted differences cannot be attributed to the construct alone.

Our goal is accordingly twofold. First, we aim to characterise how the two dominant shoulder replacement strategies differ, as used in national practice, in terms of the timing and reported indications for the first revision. Second, we aim to derive from the NJR’s separately published estimates the between-construct quantities it does not itself tabulate—the absolute difference in cumulative revision at each reported time point, with approximate confidence intervals (CIs), and the relative rates of each reported revision indication—quantifying a contrast the NJR describes only qualitatively. We do not attempt to establish which construct performs better, which would require adjusted, indication-matched, patient-level data.

Using the most recent published NJR data, we describe the cumulative probability and reported indications for the first revision after elective anatomic TSR and RTSR. We report hemiarthroplasty, which accounted for 3.6% of all primary shoulder procedures recorded in the NJR in 2024 [13], and age- and sex-stratified estimates for all elective procedures for context only. The NJR’s published outputs report revision by construct but not by primary indication within construct, so our analysis describes the constructs as used across their indications; an indication-stratified comparison would require patient-level data (Section 4.6). We did not aim to make adjusted or causal comparisons, and we distinguish the revision of primary constructs from outcomes after revision, which these data do not address.

## 2. Materials and Methods

### 2.1. Design and Reporting

We performed a secondary descriptive analysis of aggregate statistics published in the NJR’s 22nd Annual Report [13]. Reporting was informed, where applicable, by the Strengthening the Reporting of Observational Studies in Epidemiology (STROBE) statement [15] and its Reporting of Studies Conducted using Observational Routinely collected Data (RECORD) extension [16]. Because this study synthesises published aggregate statistics rather than patient-level data, item-by-item checklist compliance is neither claimed nor applicable. The NJR permits quoting statistics, charts and tables published in its Annual Reports, provided the NJR, the period, procedures and any applied filters are stated [13]. The figures presented herein are derived from published tabulated values rather than reproduced directly. The survival and incidence estimates presented herein were generated by the NJR rather than by us [13]; consequently, no adjusted, matched or multivariable analysis was possible, and all comparisons are unadjusted and descriptive.

### 2.2. Data Source, Participants and Constructs

The NJR collects data on shoulder replacements performed in England, Wales, Northern Ireland, the Isle of Man and Guernsey and has recorded shoulder procedures since 2012 [13]. However, for the revision analyses conducted herein, the NJR’s published cohort flow diagram (Figure 3.S1 [13]) applies a sequence of exclusions—for consent, tracing and valid identifiers, concordant date and patient information, jurisdiction, duplicate records, inconsistent operative patterns and procedures whose first recorded operation was itself a revision—that restricts the revision-analysis cohort to English and Welsh procedures; Northern Ireland, Isle of Man, and Guernsey records (724 of 96,945 procedures, 0.75%) are excluded before the primary-procedure revision-analysis cohort of 86,882 is formed [13]. Therefore, the analytic cohort represents England and Wales rather than all countries recorded in the NJR. The procedures analysed were performed between April 2012 and 31 December 2024, spanning the entire data collection period for shoulder procedures, and are reported in the NJR’s 22nd Annual Report, which includes records submitted before 1 March 2025 [13]. The unit of observation is the individual primary shoulder replacement; patients with bilateral procedures contribute two observations, and the NJR survivorship estimates assume that survivorship of one side is independent of the other [13]. The two index constructs are elective anatomic (conventional) TSR (*n* = 21,180) and RTSR (*n* = 39,408); proximal humeral hemiarthroplasty (*n* = 9069) is shown for context. We restricted the analysis to elective procedures because the NJR publishes construct-specific survival and revision-indication estimates separately for elective and acute-trauma procedures, and the acute-trauma population differs materially (predominantly RTSR for fracture, with 12-year cumulative mortality of 56.3% versus 42.8% after elective surgery; Tables 3.S4 and 3.S20 [13]); finer stratification by primary indication is not published (Section 4.6). The RTSR denominator is 39,408 in the NJR survival table (Table 3.S8 [13]) and 39,406 in its demographic table (Table 3.S2 [13]); this two-case difference is present in the source; all survival-based analyses (Section 3.2 and Section 3.5) and the 60,588 total use 39,408, whereas the demographic description (Section 3.1) uses 39,406 [13]. The PTIRs (Table 3.S13 [13]) are expressed per prosthesis year rather than per procedure (Section 2.3). The NJR classified the constructs using whole-construct validation; 8.6% of all procedures were unconfirmed and are not analysed as index constructs here [13].

### 2.3. Outcomes and Analysis

The primary outcome was first revision. Revision denotes any addition, removal or modification of constructs, including debridement and implant retention (DAIR) with construct exchange, excision arthroplasty, amputation and conversion to arthrodesis [13]. Procedures that do not add, remove or modify an implant—for example isolated rotator cuff repair, tendon transfer, capsular or other soft-tissue procedures, manipulation, or superficial wound debridement—fall outside the NJR definition of revision and are not counted as revision events in these analyses, with the exception of DAIR as described below; the NJR introduced a separate reoperation form only with MDS version 8, and the 69 reoperation records in the shoulder dataset are excluded from the NJR’s analytic cohort before analysis (Figure 3.S1 [13]). The NJR’s recording of DAIR without modular component exchange has changed across minimum data set (MDS) versions: it was not captured before version 7, is recorded as a revision procedure on version 7 forms, and is classified as a reoperation from version 8 onwards [13]. The NJR’s Annual Report analyses used herein (Tables 3.S8 and 3.S13 [13]) follow each primary replacement to its first revision and treat all the procedure types above, including DAIR without modular exchange recorded on MDS version 7 forms, as that revision; only the NJR’s separate surgeon-, hospital- and construct-performance monitoring excludes DAIR without exchange [13]. Because DAIR without exchange was not captured before June 2018 and has been recorded as a reoperation since June 2023, the revision endpoint is not fully homogeneous across the study period (2012–2024).

The secondary outcome was the reported indication for revision (infection, instability/dislocation, cuff insufficiency, aseptic loosening/lysis, periprosthetic fracture or other); these indications are recorded on the NJR revision form, are not mutually exclusive and are not recorded for some revisions, so they represent reported indications rather than adjudicated causes [13]. The NJR reports the cumulative probability of first revision as 1 − Kaplan–Meier estimate with 95% CIs, treating death as non-informative censoring; therefore, these estimates represent the net probability of revision assuming the patient remains alive, rather than the real-world cumulative incidence of revision before death [13,17]. Reported indications are expressed as the prosthesis-time incidence rate (PTIR, revisions per 100 prosthesis years at risk). The PTIRs (Table 3.S13 [13]) derive from the same elective revision-analysis cohort, revision definition and administrative censoring date (31 December 2024) as the cumulative revision estimates (Table 3.S8 [13]), with 851 first revisions over 118,730 prosthesis years for anatomic TSR and 1077 over 168,260 prosthesis years for RTSR, corresponding to a mean of approximately 5.6 and 4.3 years at risk per procedure, respectively. Because RTSR was adopted later, its pooled PTIRs are weighted toward earlier follow-up, so indications that arise late are relatively under-represented for RTSR. The NJR flags cumulative revision estimates (Table 3.S8 [13]) as imprecise when ≤250 procedures remain at risk; this rule does not apply to the PTIRs.

### 2.4. Derived Between-Construct Analyses

The registry’s commentary—framed around stemmed anatomic TSR (the NJR’s ‘conventional total shoulder replacement’) versus stemmed RTSR constructs, whose estimates closely match the all-construct values—describes an initially higher revision rate after RTSR that then plateaus, a more steadily rising rate after anatomic TSR, and an overall difference of about 3.5 percentage points at 12 years [13]; however, it does not tabulate the between-construct difference at each reported time point with 95% CIs, nor the ratio of indication-specific revision rates. We derived these quantities from the published point estimates and their 95% CIs (Tables 3.S8 and 3.S13 [13]); they constitute the analytic contribution of this study.

First, at each reported time point we calculated the absolute difference in cumulative revision, D = p_A_ − p_R_, where p_A_ and p_R_ are the published 1 − Kaplan–Meier estimates (percentage scale) for anatomic TSR and RTSR. For each construct, we recovered an approximate standard error (SE) from its published 95% CI, SE_i_ = (U_i_ − L_i_)/(2 × 1.96), where L_i_ and U_i_ are the lower and upper limits. The SE of the difference was SE_D_ = √(SE_A_^2^ + SE_R_^2^), and the approximate 95% CI was D ± 1.96 × SE_D_. The covariance term is zero by design because the two constructs are disjoint cohorts whose estimates share no observations.

Second, for each reported indication we calculated the ratio of PTIRs, RR = PTIR_R_/PTIR_A_, with SE(ln PTIR_i_) = (ln U_i_ − ln L_i_)/(2 × 1.96) and approximate 95% CI exp[ln RR ± 1.96 × √(SE_A_^2^ + SE_R_^2^)] on the log scale; where a published lower limit was rounded to 0, we calculated the point ratio but could not recover a finite CI. We describe these intervals as approximate for three reasons: the published estimates and limits are rounded to two decimal places; the NJR does not state the transformation scale on which its Kaplan–Meier intervals were computed, and we treated each interval as symmetric on the percentage scale, whereas intervals computed on a transformed scale are slightly asymmetric; and a normal approximation was applied to the difference. In a sensitivity check using, for each construct, the larger of the two half-widths of its published interval, no derived limit moved by more than 0.08 percentage points. All derived quantities are unadjusted.

These derived quantities describe the between-construct contrast that the NJR qualitatively reports but does not tabulate; they do not adjust for differences in indications, ages or case-mix between the two constructs and are interpreted with the confounding caveats outlined in Section 4.6 and Section 4.9.

We performed statistical analyses using Python (version 3.13; Python Software Foundation, Wilmington, DE, USA) with the NumPy (version 2.5) and SciPy (version 1.18) libraries, and constructed the figures from the NJR’s published values using Matplotlib (version 3.11).

## 3. Results

### 3.1. Case-Mix and Shift in Practice

Among elective procedures, patients who underwent RTSR were older (median 76 years, interquartile range [IQR] 70–80) than those who underwent anatomic TSR (median 69 years, IQR 62–75) [13]. The primary indication for anatomic TSR was osteoarthritis (19,780 of 21,180, 93.4%). Cuff tear arthropathy was treated almost exclusively by RTSR (17,531 of 19,775 such procedures, 88.7%) [13]. Osteoarthritis was nonetheless also a recorded indication for 16,344 RTSR procedures (41.5% of 39,406; indications are not mutually exclusive), so the RTSR cohort comprises both cuff-deficient and cuff-intact osteoarthritic shoulders (Table 3.S4 [13]). This confounding by indication—patients who underwent RTSR were a median of 7 years older—is fundamental to interpreting every comparison that follows. Over the study period, the preferred procedure inverted: as a proportion of all primary shoulder procedures (elective and acute trauma) recorded in the NJR, RTSR increased markedly from 24.0% in 2012 to 70.4% in 2024, while hemiarthroplasty decreased markedly from 39.7% to 3.6%, and anatomic TSR decreased modestly from 22.5% to 18.8% (Table 1) [13].

### 3.2. Timing of Revision

**Figure 1:**
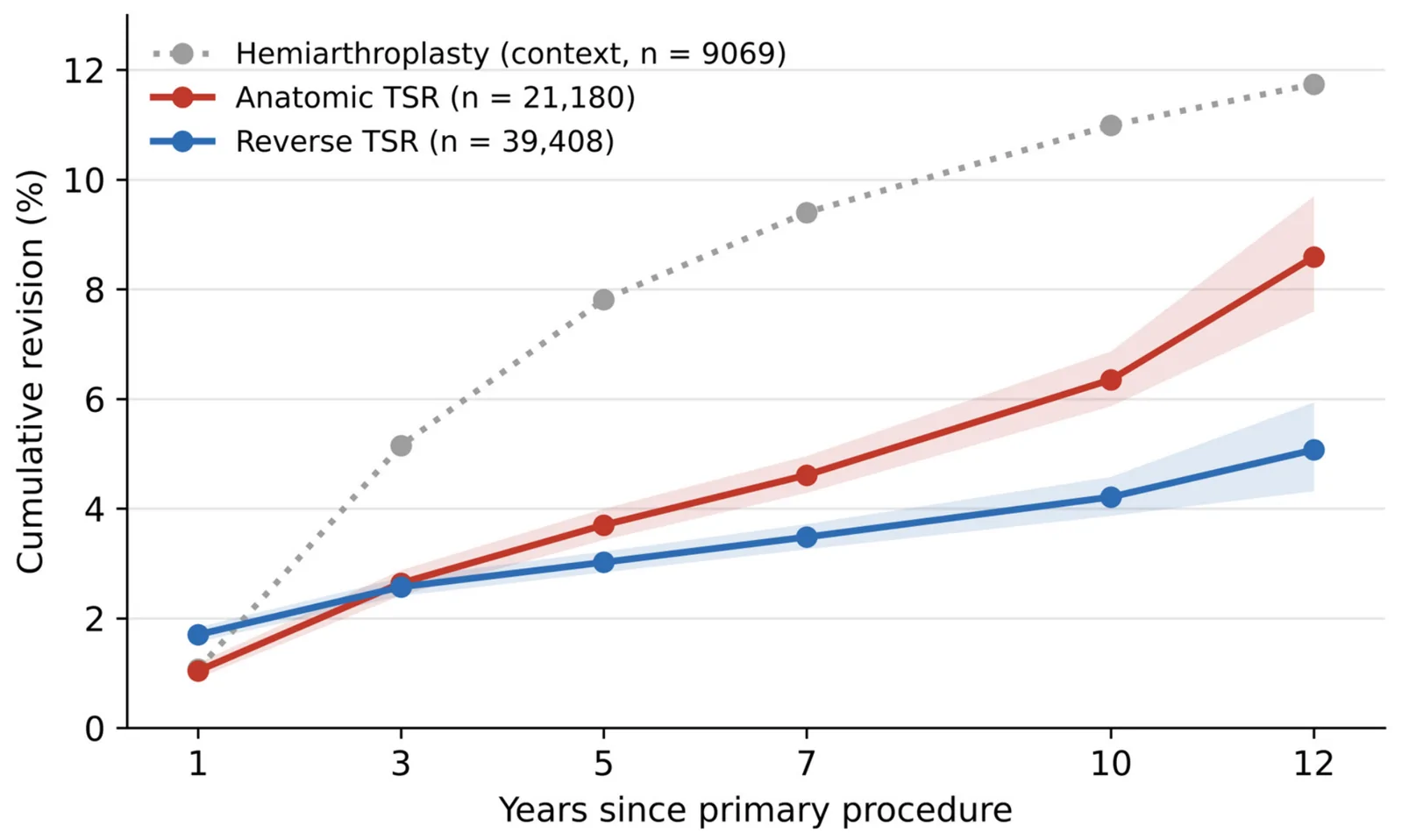
Cumulative revision after elective anatomic TSR and RTSR. Constructed from Table 3.S8 [13]. Markers show the reported 1 − Kaplan–Meier estimates and shaded bands their 95% CIs; straight lines join reported time points and are a visual aid only, not the underlying Kaplan–Meier curves. The ordering of the anatomic TSR and RTSR estimates reverses between the 1- and 3-year time points. Hemiarthroplasty is shown in grey for context.

No single time point captures the central finding (Table 2, Figure 1). At 1 year, the cumulative revision estimate was higher for RTSR (1.70%, 95% CI [1.57, 1.84]) than for anatomic TSR (1.04%, 95% CI [0.91, 1.19]). By 3 years, the cumulative revision estimate was instead higher for anatomic TSR (2.64%, 95% CI [2.42, 2.88]) than for RTSR (2.57%, 95% CI [2.41, 2.74]), although the approximate interval for the difference included 0 (Section 3.5). By 5 years, the cumulative revision estimate had reached 3.70% (95% CI [3.43, 4.00]) for anatomic TSR compared with 3.02% (95% CI [2.84, 3.22]) for RTSR. By 12 years, the cumulative revision estimate had reached 8.59% (95% CI [7.60, 9.70]) for anatomic TSR compared with 5.07% (95% CI [4.32, 5.94]) for RTSR.

The trajectories also differ for anatomic TSR and RTSR. For RTSR, the 1-year cumulative revision estimate (1.70%) is about one-third of its 12-year estimate (5.07%); between 5 and 12 years, the estimate increased by only 2.05 percentage points [13]. For anatomic TSR, the cumulative revision estimate increased more slowly at first but steadily, adding 4.89 percentage points between 5 and 12 years (Figure 1). Because the published data provide only selected point estimates from step-function Kaplan–Meier curves, we cannot recover the exact time the order switched; therefore, we state only that the ordering reverses between the 1- and 3-year cumulative revision estimates.

### 3.3. Caution When Comparing These Estimates

These are unadjusted cumulative revision estimates. Two cautions apply before they can be interpreted as construct performance. First, the two constructs were used for different indications in populations that differed systematically at baseline (patients who underwent RTSR were a median of 7 years older). Second, because revision is a treatment decision rather than a purely biological endpoint, the estimates partly reflect surgical decisions as well as construct behaviour. Section 4.6 and Section 4.9 address these issues in more detail.

### 3.4. Reported Indications for Revision

**Figure 2:**
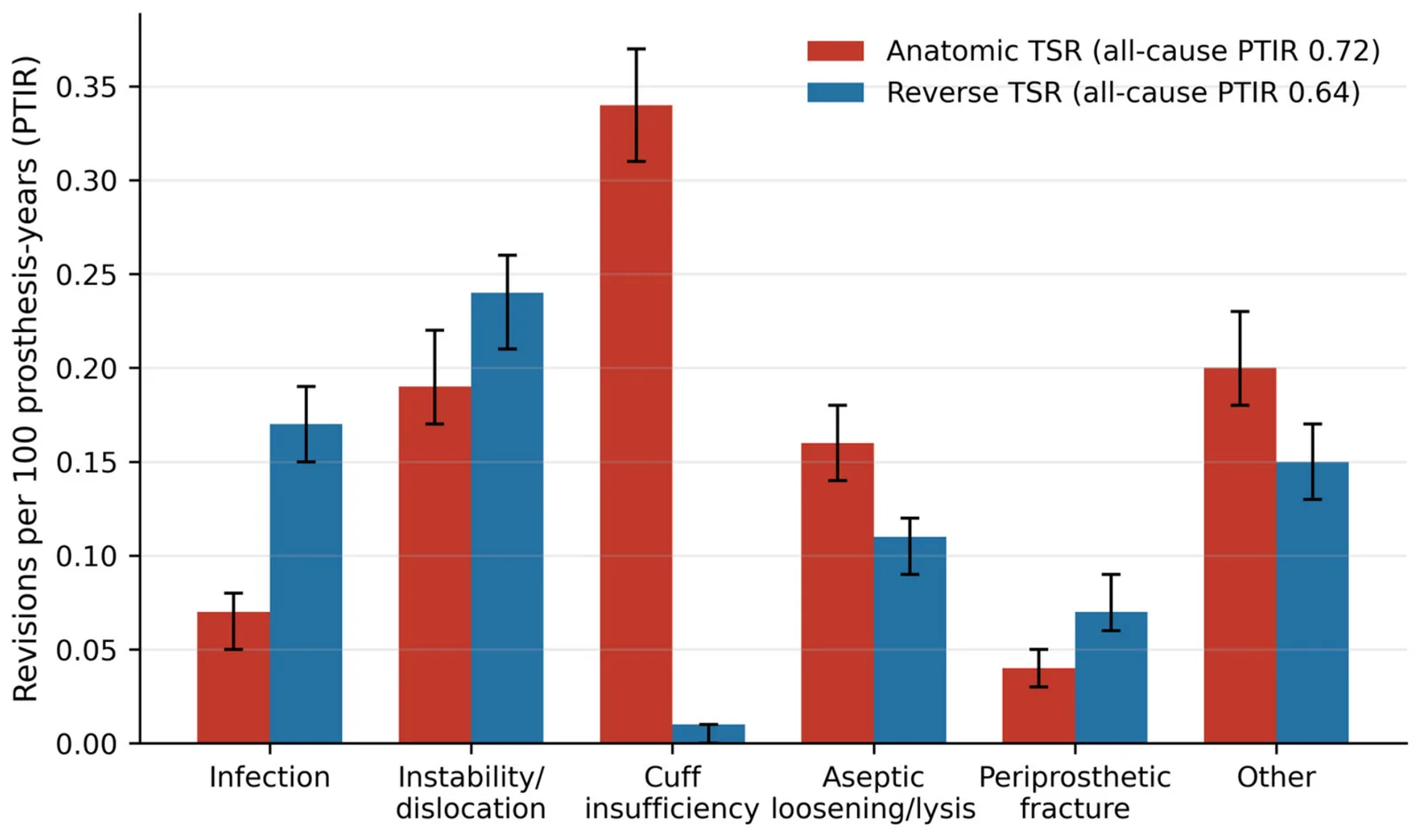
Reported indication for first revision after elective anatomic TSR and RTSR. Constructed from NJR Table 3.S13 [13]. Bars show PTIR point estimates with 95% CI error bars. Indications are not mutually exclusive.

The reported indications for revision differed by construct (Figure 2; Supplementary Table S1) [13]. Cuff insufficiency was the dominant indication for revision after anatomic TSR (PTIR 0.34, 95% CI [0.31, 0.37]) but was rarely the indication for revision after RTSR (PTIR 0.01, 95% CI [0.00, 0.01]). In contrast, instability/dislocation was the dominant indication for revision after RTSR (PTIR 0.24, 95% CI [0.21, 0.26]), modestly higher than after anatomic TSR (PTIR 0.19, 95% CI [0.17, 0.22]). Infection was recorded as the indication more than twice as often after RTSR (PTIR 0.17, 95% CI [0.15, 0.19]) than after anatomic TSR (PTIR 0.07, 95% CI [0.05, 0.08]). The NJR does not report between-construct inferential tests for these indications; therefore, we derived rate ratios between constructs, which are reported separately in Section 3.5.

Because these indication profiles are pooled across the entire study period rather than stratified by time since surgery, they describe the constructs’ overall reported indication profiles but do not indicate when each indication arose; we discuss their interpretation in Section 4.2 and Section 4.3.

### 3.5. Derived Between-Construct Comparison

**Figure 3:**
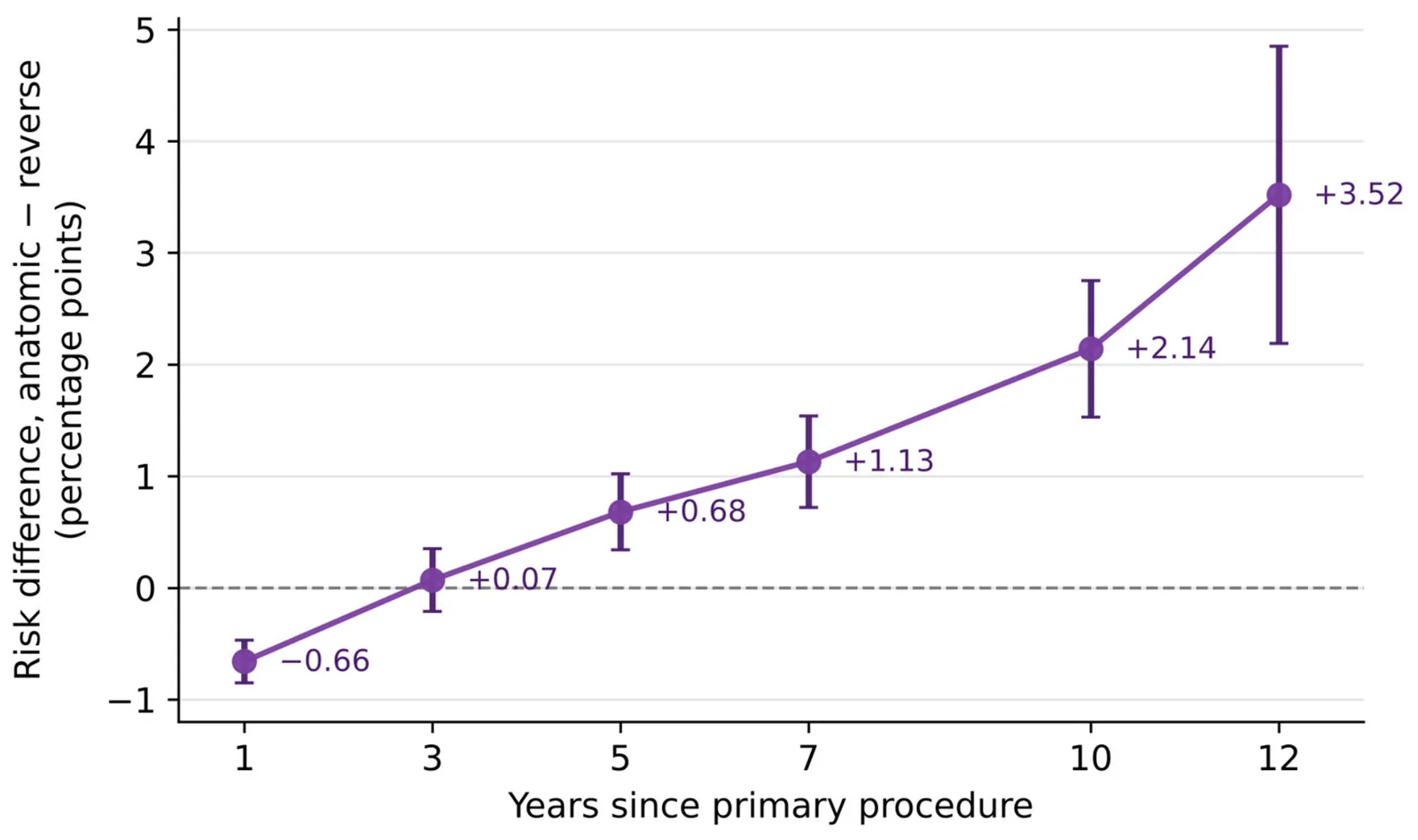
Derived between-construct difference in cumulative revision (anatomic TSR minus RTSR) with 95% CIs. Author-derived from Table 3.S8 [13]. Below 0, RTSR has the higher cumulative revision estimate; above 0, anatomic TSR does. The difference crosses 0 between the 1- and 3-year estimates.

The NJR describes the between-construct pattern qualitatively (for stemmed constructs) but does not tabulate the difference at each time point [13]. Deriving that difference (anatomic TSR minus RTSR) from the published estimates makes it explicit (Table 3, Figure 3). At 1 year, the difference was negative (−0.66 percentage points, approximate 95% CI [−0.85, −0.47]), reflecting more frequent early revision after RTSR. At 3 years, the difference was close to 0 (0.07, 95% CI [−0.21, 0.35]). After 5 years, it became positive and widened progressively from 0.68 (95% CI [0.34, 1.02]) at 5 years to 2.14 (95% CI [1.53, 2.75]) at 10 years and 3.52 (95% CI [2.19, 4.85]) at 12 years. These unadjusted differences describe the two constructs as used in different populations and should not be read as evidence that either construct has superior survivorship. The sign change between the 1- and 3-year estimates quantifies the inversion that is otherwise only qualitatively visible in the separate curves. The 95% CIs are approximate, as they were recovered from the NJR’s rounded published intervals; therefore, formal between-construct significance testing would require patient-level data.

Expressing the reported indications as PTIR ratios (RTSR relative to anatomic TSR) similarly quantifies the between-construct contrast (Table 4) [13]. They describe the relative frequency of each recorded indication and share the same overlap and missing-indication caveats as the underlying PTIRs. The PTIR for infection was 2.43 times higher for RTSR (95% CI [1.87, 3.16]), the PTIR for periprosthetic fracture was 1.75 times higher for RTSR (95% CI [1.26, 2.42]), and the PTIR for instability/dislocation was modestly (1.26 times) higher for RTSR (95% CI [1.07, 1.49]). Conversely, the PTIR for aseptic loosening/lysis (0.69, 95% CI [0.57, 0.83]) and the ‘other’ category (0.75, 95% CI [0.63, 0.90]) were lower for RTSR. Notably, cuff insufficiency was markedly lower for RTSR (rate ratio ≈0.03; PTIR 0.01 vs. 0.34 per 100 prosthesis years); a 95% CI could not be recovered because the rounded source interval for RTSR [0.00, 0.01] includes 0.

### 3.6. Age and Sex

**Figure 4:**
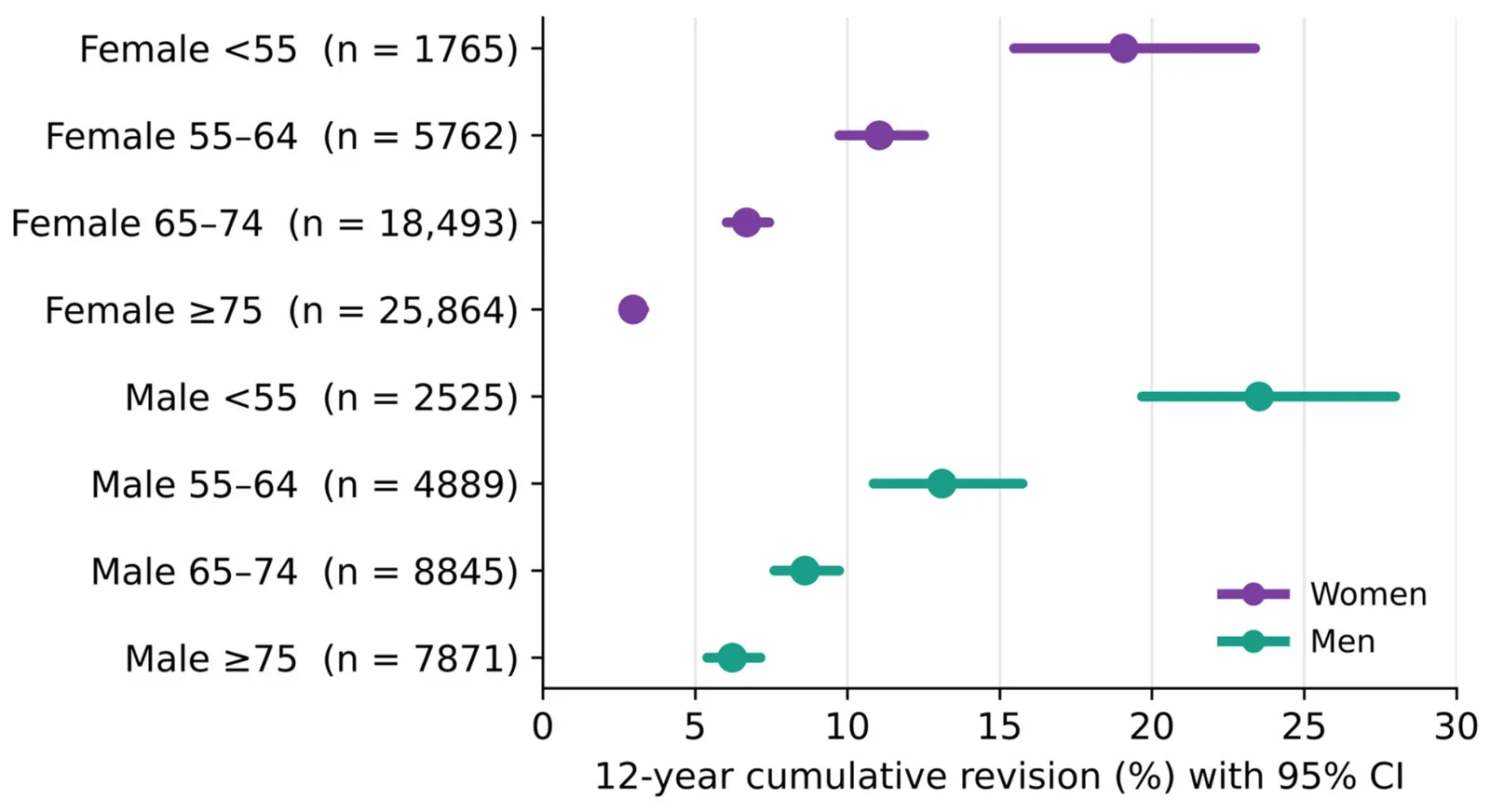
Twelve-year cumulative revision estimate by age and sex across all elective primary shoulder replacements (contextual, not construct-specific). Constructed from Table 3.S7 [13]; purple, women; green, men.

We present the NJR’s age- and sex-stratified revision estimates for all elective primary shoulder replacements (Table 3.S7 [13]) for one reason: because patients receiving RTSR were a median of 7 years older than those receiving anatomic TSR, the size of the age gradient in revision indicates how much of any between-construct difference could be produced by age alone. These estimates are not stratified by construct, describe the whole elective cohort rather than the anatomic TSR-plus-RTSR cohort that is the focus of this study, and are presented as context only (Figure 4; Supplementary Table S2). Within these all-elective estimates, the 12-year cumulative revision estimate ranged from 2.96% (95% CI [2.63, 3.33]) in women aged ≥75 years to 23.52% (95% CI [19.67, 27.98]) in men aged <55 years—an approximately eightfold difference across these age–sex strata—with estimates higher in men than in women within every age band [13]. Twelve-year revision was lowest in the ≥75-year strata, in which RTSR recipients are concentrated, and these strata also have by far the highest competing mortality (12-year cumulative mortality 62.7% in women and 70.9% in men aged ≥75 years versus 11.6% and 11.7% in those aged <55 years; Table 3.S21 [13]). Because these estimates combine constructs, they cannot establish whether the anatomic TSR versus RTSR ordering behaves differently in younger patients; that would require a construct-by-age analysis, which the published aggregate outputs do not provide.

## 4. Discussion

### 4.1. Principal Findings

Among the 60,588 elective anatomic TSR and RTSR procedures, used largely for different indications, the two constructs differed in revision timing and indication—that is, they showed different unadjusted revision profiles rather than a uniformly better or worse result for either construct. RTSR had a higher 1-year cumulative revision estimate but a lower unadjusted estimate at every subsequent reported time point. The estimate increased more steeply soon after RTSR and more slowly thereafter, but more steadily after anatomic TSR. Consequently, the ordering reversed between the 1- and 3-year estimates, and by 12 years the estimate was approximately 3.5 percentage points higher for anatomic TSR than for RTSR. The reported indications for revision differed by construct; most strikingly, cuff insufficiency was common after anatomic TSR and near-absent after RTSR. Age- and sex-stratified estimates, available only for all elective procedures combined and not by construct, ranged roughly eightfold across age–sex strata.

### 4.2. Early Revision After RTSR: Biomechanics, Instability and Infection

In the NJR data, instability/dislocation and infection were the leading reported indications for revision after RTSR (Section 3.4); the literature offers the following mechanisms. The RTSR construct medialises and distalises the glenohumeral centre of rotation, converting the superiorly directed pull of the deltoid into an elevating moment and substituting for a deficient rotator cuff [5,6,18]. This reconfiguration restores elevation but removes the concavity-compression stabilising mechanism the intact cuff provides in the native and anatomically resurfaced shoulder [19]; stability instead depends on deltoid tension, the depth and version of the glenosphere–socket articulation, and avoiding bony or construct impingement [6,18]. Therefore, instability arises when soft-tissue tension is inadequate, when components are malpositioned or when medialisation is excessive—mechanisms established at, or shortly after, the index operation—which is consistent with clinical series describing atraumatic dislocation within the first postoperative weeks [20]. A systematic review and a large clinical series identified instability as one of the most frequent complications of RTSR, with dislocation rates of about 3–5% after primary surgery [21,22]. Recognised contributors include subscapularis insufficiency or irreparability, fracture sequelae, younger age and the revision setting [22,23]. The near-absence of cuff-related revision after RTSR in our cohort (PTIR 0.01) reflects this independence from the cuff and is consistent with instability and infection, rather than cuff failure, being the leading reported indications for revision after RTSR [13].

Infection was a leading reported indication for revision after RTSR (PTIR 0.17), occurring more than twice as often as after anatomic TSR (PTIR 0.07, 95% CI [0.05, 0.08]) [13]. In broader practice, RTSR is used in older patients and in more complex reconstructions, including those following fracture, fracture sequelae and revision of failed prior arthroplasty, in which complication rates are substantially higher than after primary surgery [21,24]. Because our cohort is elective, primary fracture- and revision-related mechanisms cannot be assumed to explain this pattern. Within the arthroplasty population, deep infection has been associated with younger age, male sex, traumatic indication and RTSR arthroplasty, with *Cutibacterium* (*Propionibacterium*) *acnes* being the most commonly identified microorganism by culture [25]. Therefore, the NJR pattern may reflect the patients and indications for which RTSR is chosen as much as the construct itself; aggregate data cannot separate these factors [13].

### 4.3. Later Revision After Anatomic TSR and the Two Revision Profiles

In the NJR data, cuff insufficiency and aseptic loosening dominated the reported indications after anatomic TSR and revision accrued progressively (Section 3.2 and Section 3.4). Anatomic TSR depends on two elements that RTSR does not—a functioning rotator cuff and a durable glenoid component—and its more progressive revision profile is consistent with the gradual failure of both [26,27,28]. Secondary rotator cuff dysfunction is a recognised late complication: a multicentre series with more than 5 years of follow-up documented progressive cuff dysfunction and superior humeral head migration developing over time, with associated deterioration in function [28]. As the cuff fails, eccentric loading is transmitted to the glenoid, generating the ‘rocking-horse’ loosening mechanism long associated with glenoid failure, and glenoid complications feature prominently in reviews and clinical series of anatomic arthroplasty failure [26,27,29]. A contemporary systematic review found construct loosening, chiefly of the glenoid, to be the leading failure mode overall (explaining around a quarter of failures) and the dominant mode beyond 5 years, with rotator cuff insufficiency the next most frequent [30]. These time-dependent processes offer a biologically plausible—though, from NJR data alone, unproven—explanation of why revision accrues progressively after anatomic TSR [13,30].

Our study’s two principal observations differ in their vulnerability to confounding by indication. The construct-specific pattern of reported indications is plausibly the less confounded: that cuff insufficiency is recorded far more often after anatomic TSR than after RTSR follows from the constructs’ differing dependence on the rotator cuff, although diagnosis, coding, revision threshold and construct selection may also contribute and cannot be disentangled in aggregate data. Because timing is far more vulnerable to confounding (Section 4.6), we place more weight on the reported differences in indication. Within that frame, the wider literature associates RTSR with early, largely mechanical modes of revision (instability, infection, component or baseplate problems) and anatomic TSR with progressive, degenerative modes (cuff attrition, glenoid loosening or polyethylene wear) that accumulate with time [24,27,28,30]. Our data are consistent with this framing, but show indications pooled across follow-up, so they cannot establish that a given cause acted early or late; a single revision percentage at one time point therefore conflates two different revision profiles.

### 4.4. The Age and Sex Gradients

The age gradient bears directly on the primary comparison: an unadjusted contrast between a cohort with a median age of 69 years and one with a median age of 76 years will differ substantially on age alone, in the direction of lower revision for the older (RTSR) cohort. The eightfold age gradient we observed across all elective procedures is consistent with patterns seen across joint replacement and specifically in shoulder replacement, where long-term series of patients aged ≤50 years reported lower construct survival and high rates of unsatisfactory results [11,13,31]. Several non-exclusive explanations are plausible: younger patients impose higher mechanical demands on the construct, accelerating polyethylene wear and mechanical loosening; they live much longer following replacement, increasing the probability of a revision event; and they may have a lower threshold for undergoing revision, being fitter for surgery and having higher functional expectations [11]. Differing indications may compound this if younger patients more often undergo replacement for sequelae of trauma, inflammatory arthritis or instability; however, the aggregate data are not jointly stratified by age and indication, so this remains a possible rather than a demonstrated explanation. The consistent excess revision in men within every age band is more difficult to attribute and should be interpreted cautiously: it may partly reflect higher activity or occupational demand, differences in indication and body habitus, or the higher infection risk in men reported in some shoulder cohorts, rather than any intrinsic biological difference the NJR can identify [25].

### 4.5. Comparison with the Wider NJR and Clinical Literature

Our findings are broadly concordant with, and extend, the existing evidence. A propensity-matched NJR/HES comparison of RTSR and anatomic TSR for osteoarthritis with an intact cuff provides the adjusted counterpart to our unadjusted description; in patients aged ≥60 years, it found RTSR acceptable and did not demonstrate a clinically important long-term difference in revision between constructs (maximum follow-up of 8.75 years) [9]. The near-universal contemporary use of RTSR for cuff tear arthropathy, and its increase to 70.4% of all primary shoulder procedures (England and Wales) recorded in the NJR, are consistent with the international trend described in a meta-analysis of 11 public registries, which also reported RTSR and anatomic TSR used in near-equal proportions for osteoarthritis despite limited high-quality comparative evidence [7].

An analysis of the Australian Orthopaedic Association National Joint Replacement Registry offers an instructive parallel from the revision setting: analysing the second revision after the aseptic first revision, it found a markedly higher risk of second revision for RTSR constructs in the first month only (hazard ratio 4.8, 95% CI [2.2, 9]), after which the risk equalised, with a cumulative second revision rate of 24% for RTSR compared with 20% for non-RTSR at 8 years [32]. Although drawn from a different revision setting and comparator, that early-then-convergent pattern offers an indirect parallel to the early concentration of revision we observe after primary RTSR surgery. A long-term single-cohort follow-up of Grammont-style RTSR reported 10-year construct survival of about 93% but continuing complications and a decline in functional scores between medium- and long-term review [33], so the plateau of the curve within 12 years in the NJR should not be extrapolated further. The clinical significance of a first revision not being a definitive solution is emphasised by an NJR/HES analysis of re-revision after revision of a failed primary RTSR, which found further revision common and instability as the first-revision indication associated with increased re-revision risk [34]. Altogether, these sources caution against a simple ‘RTSR is more durable’ interpretation: differences across studies are best explained by follow-up horizon, case-mix and adjustment rather than by any single construct being uniformly superior [7,9,30].

### 4.6. Confounding by Indication: The Central Interpretive Limit

The main threat to any comparison of these constructs is that they are not two treatments for the same condition. Cuff tear arthropathy was treated almost exclusively by RTSR, anatomic TSR was used almost exclusively for osteoarthritis, and the RTSR cohort was a median of 7 years older. Therefore, in our data, differences in revision between constructs are inseparable from differences in underlying disease, rotator cuff status, and patient age. Crucially, the principal indication in which the two constructs compete for the same patient—osteoarthritis with an intact cuff—has been examined in an adjusted, propensity-matched NJR/HES analysis, which found no clinically important long-term difference in revision between RTSR and anatomic TSR among patients aged ≥60 years [9]. That an adjusted, indication-matched comparison finds little difference, while our unadjusted whole-cohort comparison finds a sizeable one, is itself consistent with the whole-cohort difference being driven substantially by indication and age rather than by construct. Therefore, we interpret our timing findings as describing how the two constructs behave as used in practice and defer to the matched analysis for the question of construct effect within the shared indication [9]. A comparison restricted to osteoarthritis with an intact rotator cuff would be the most informative test of construct effect. However, the NJR’s published shoulder outputs stratify cumulative revision by construct, by age and sex, and by brand, but not by primary indication within construct (Tables 3.S6–3.S10 [13]); such an analysis therefore cannot be performed from aggregate data and would require a patient-level data application to the registry. We regard this as a major limitation of the present study.

### 4.7. Evolving Indications, Construct Design and Surgical Practice

Several secular trends impact interpretation. The indication for RTSR has expanded from cuff tear arthropathy to osteoarthritis with an intact cuff, fracture and revision. Thus, the contemporary RTSR population is heterogeneous and differs from the cuff-arthropathy population in which the construct was first validated. Expanding indications may alter survivorship in ways that a pooled 2012–2024 analysis cannot resolve [7]. Construct design has also evolved over the same period, including toward lateralised centres of rotation and modified glenosphere configurations intended to reduce notching and improve stability. Although such changes are biologically plausible modifiers of early instability, the NJR cannot attribute changes in revision to specific design features [6,18]. Surgeon and unit volume also matter: the NJR/HES volume analysis demonstrated a revision threshold of around 10 procedures per year, and the median consultant volume recorded by the NJR, though increasing, remains modest (median 14 procedures per consultant in 2024; Supplementary Note S1) [12,13]. One hypothesis, not tested here, is that a learning curve for a technically demanding procedure contributes to early revision; our data include no surgeon-experience analysis and cannot isolate a learning effect from other early revision mechanisms.

### 4.8. Implications for Clinical Practice

These data may inform, but should not by themselves determine, construct selection. They describe how revision risk is distributed over time and by reported indication for two constructs used in systematically different populations and cannot establish that one construct is preferable for a patient of a given age (Section 4.6). Because follow-up does not extend beyond 12 years and the late estimates are imprecise (Section 4.9), short-term revision estimates should not be extrapolated to a patient’s full exposure period, and the NJR cannot determine whether the difference at 12 years would persist, widen or narrow with longer follow-up [13]. In practice, construct choice depends on factors this analysis does not capture: indication, rotator cuff integrity, glenoid morphology, bone quality, chronological and biological age, comorbidity and infection risk, and the difficulty and outcomes of revising each construct should it fail [9,30,34]. The appropriate use of these findings is to support shared decision-making—making explicit that the constructs differ in the timing and reported indications for revision—alongside adjusted, indication-specific evidence, rather than to prescribe a construct by age. We deliberately propose neither a threshold age nor a rule because unadjusted descriptive data cannot support one.

### 4.9. Strengths and Limitations

Our study has several strengths that lend weight to its findings. First, it draws on a large, prospectively collected national registry dataset capturing real-world multicentre practice across England and Wales, minimising the selection bias inherent to single-centre series and yielding precise estimates for the two common constructs [13]. Second, the 12-year follow-up allows examination of the trajectory of revision, rather than a single time point, showing that the ordering of the two constructs changes over time, and whole-construct validation improves confidence in construct classification. Third, presenting cumulative revision alongside the reported indication for revision makes explicit that the two constructs differ in both the timing and reported indications for revision, a description not apparent from either factor read alone.

However, our study also has several limitations. First and foremost, because only published aggregate data were available, no adjustment was possible: no multivariable, matched, competing risks or interaction analyses could be performed. Second, the only between-construct quantities are the author-derived absolute differences in cumulative revision and PTIR ratios, whose 95% CIs are approximate (recovered from rounded published 95% CIs) and unadjusted, so any apparent difference between constructs may reflect case-mix—including the confounding by indication described in Section 4.6 and probable differences in comorbidity and bone quality—rather than construct behaviour, and none of the comparisons can be interpreted causally; adjusted, matched analyses should be preferred where available.

Third, interpretation is further constrained by how revision is measured and counted. Revision is a decision, not simply an event, and an imperfect proxy for clinical failure [1]: it undercounts failure where frail or low-demand patients with poorly functioning replacements are managed non-operatively, and may overcount it in younger, fitter patients who are more readily offered, and accept, revision for comparable dysfunction. Because RTSR is used in an older population and anatomic TSR in a younger one, these opposing biases need not cancel, although their net direction cannot be determined from aggregate data.

Fourth, death is a competing event: the 1 − Kaplan–Meier estimates give the net probability of revision assuming survival, which exceeds the real-world cumulative incidence of revision before death [17]. This is not a minor concern: the NJR reports 12-year cumulative mortality of 42.8% after elective primary shoulder replacement, rising to 62.7% in women and 70.9% in men aged ≥75 years, compared with about 12% in patients aged <55 years (Tables 3.S20 and 3.S21 [13]). Mortality is not published by construct, but because RTSR recipients are concentrated in the older strata, the gap between the net estimate and the cumulative incidence is expected to be larger for RTSR than for anatomic TSR; a competing-risks analysis would require patient-level data. Fifth, because the handling of DAIR without construct exchange changed across MDS versions (Section 2.3), the revision endpoint is not fully homogeneous over the study period. Such procedures counted as revisions only on MDS version 7 forms (June 2018 to June 2023), so revision—and infection-attributed revision in particular—was ascertained more completely within that window; because RTSR formed a growing majority of procedures in those years, any such effect would tend to inflate early revision and infection PTIRs for RTSR relative to anatomic TSR. The NJR does not publish the number of such procedures, so the magnitude cannot be quantified. Sixth, because reported indications overlap and are sometimes absent, the PTIRs represent a lower bound on attribution rather than a partition of causes. Seventh, this study addressed revision-free construct survivorship only; it did not assess functional success, radiographic failure, pain or patient satisfaction, which are distinct endpoints that the NJR reports separately [1,13].

Finally, several cohort features limit the generalisability and precision of our findings. Because construct designs, glenosphere configurations, surgical technique and indications all changed materially between 2012 and 2024, pooled estimates may not reflect contemporary practice. Follow-up is also strongly unbalanced between constructs, as RTSR utilisation increased from 24.0% to 70.4% of primary procedures over the study period, so the late estimates for both constructs derive from earlier-era constructs and rest on small numbers at risk, with secular change a particular concern for RTSR. Moreover, because whole-construct validation left 8.6% of constructs unconfirmed, which were disproportionately RTSR (4952 of 7473, 66.3%, versus 53.6% of all procedures) [13], excluded RTSR cases could bias the RTSR estimates in an undetermined direction. Furthermore, the aggregate data allow no adjustment for surgeon or hospital despite a demonstrated volume–revision relationship [12]. Therefore, this study is descriptive and hypothesis-generating rather than a comparative-effectiveness analysis and cannot establish that either construct produces a different outcome.

## 5. Conclusions

Across 60,588 elective anatomic TSR and RTSR procedures recorded in the NJR and performed in England and Wales between 2012 and 2024, the two constructs, as used for largely different indications and in patients of different ages, showed different revision profiles in both the timing of revision and its reported indications. The most robust observation is the construct-specific pattern of reported indications: cuff insufficiency was a leading indication for revision after anatomic TSR but was near-absent after RTSR, a pattern consistent with the RTSR construct’s independence from the rotator cuff; however, the aggregate data do not allow determination of how much of this difference reflects construct design rather than case-mix or coding. The difference in the timing of revision—a higher early estimate after RTSR, with the anatomic TSR estimate rising more steadily, the ordering reversing between the 1- and 3-year estimates, and the anatomic TSR estimate about 3.5 percentage points higher by 12 years—cannot be attributed to the constructs alone. In the principal indication where the constructs are used interchangeably, an adjusted registry analysis of patients aged ≥60 years found no clinically important long-term difference [9]. As an unadjusted secondary analysis of published aggregate data, our study cannot establish the superiority of either construct or support a construct selection rule. Its contribution is descriptive and hypothesis-generating: it quantifies how revision and its reported indications are distributed for the two dominant procedures in national practice. Overall, our findings highlight the urgent need for an adjusted, patient-level, indication-matched comparison of anatomic TSR and RTSR in younger patients with osteoarthritis and an intact cuff, ideally incorporating patient-reported outcomes and extending beyond the currently available follow-up, together with routine publication by the registry of construct survival stratified by primary indication.

## Figures and Tables

**Table 1:** Utilisation and case-mix for elective anatomic TSR and RTSR (hemiarthroplasty shown for context).

Construct	*N* (Table 3.S2 [13])	Male, *n* (%)	Median Age (IQR)	% of All Primary Procedures in 2012	% of All Primary Procedures in 2024
Anatomic TSR	21,180	7235 (34.2)	69 (62–75)	22.5	18.8
RTSR	39,406	11,722 (29.7)	76 (70–80)	24.0	70.4
Hemiarthroplasty (context)	9069	3135 (34.6)	70 (60–77)	39.7	3.6

Sources: *N*, sex and age are taken from Table 3.S2 [13] (elective cases), and utilisation percentages are taken from Table 3.S1 [13], which covers all primary procedures (elective and acute trauma), so the 24.0% and 70.4% values are proportions of all primary procedures rather than elective-only. The RTSR denominator is 39,408 in the revision-analysis table (Table 3.S8 [13]) and 39,406 in the demographic table (Table 3.S2 [13]); this two-case difference is present in the source report, and the 60,588 elective total uses 39,408 [13]. RTSR, reverse total shoulder replacement; TSR, total shoulder replacement; IQR, interquartile range.

**Table 2:** Cumulative percentage revision (95% CI) after elective anatomic TSR and RTSR.

Construct	*N* (Table 3.S8 [13])	1 Year	3 Years	5 Years	7 Years	10 Years	12 Years
Anatomic TSR	21,180	1.04 [0.91, 1.19]	2.64 [2.42, 2.88]	3.70 [3.43, 4.00]	4.61 [4.29, 4.96]	6.35 [5.87, 6.87]	8.59 [7.60, 9.70]
RTSR	39,408	1.70 [1.57, 1.84]	2.57 [2.41, 2.74]	3.02 [2.84, 3.22]	3.48 [3.26, 3.72]	4.21 [3.87, 4.58]	5.07 [4.32, 5.94]
Hemiarthroplasty (context)	9069	1.07 [0.88, 1.31]	5.15 [4.70, 5.65]	7.81 [7.24, 8.42]	9.40 [8.77, 10.09]	10.99 [10.26, 11.77]	11.74 [10.90, 12.64]

Source: NJR Table 3.S8 [13]. CI, confidence interval.

**Table 3:** Derived absolute difference in cumulative revision (anatomic TSR minus RTSR) from Table 3.S8 [13] estimates.

Time	Anatomic TSR %	RTSR %	Difference (pts)	95% CI
1 year	1.04	1.70	−0.66	[−0.85, −0.47]
3 years	2.64	2.57	0.07	[−0.21, 0.35]
5 years	3.70	3.02	0.68	[0.34, 1.02]
7 years	4.61	3.48	1.13	[0.72, 1.54]
10 years	6.35	4.21	2.14	[1.53, 2.75]
12 years	8.59	5.07	3.52	[2.19, 4.85]

All values were derived by the authors from Table 3.S8 point estimates and 95% CIs [13], not reported by the NJR. Positive values indicate higher cumulative revision after anatomic TSR. The 95% CIs are approximate, as they were recovered from the NJR’s rounded published intervals assuming independent construct estimates; no formal significance test is presented. Estimates are unadjusted.

**Table 4:** Derived ratio of reported indication PTIRs (RTSR relative to anatomic TSR) from Table 3.S13 [13].

Reported Indication	Anatomic TSR	RTSR	Ratio (RTSR/Anatomic TSR) [95% CI]
Infection	0.07	0.17	2.43 [1.87, 3.16]
Instability/dislocation	0.19	0.24	1.26 [1.07, 1.49]
Periprosthetic fracture	0.04	0.07	1.75 [1.26, 2.42]
Aseptic loosening/lysis	0.16	0.11	0.69 [0.57, 0.83]
Other	0.20	0.15	0.75 [0.63, 0.90]
Cuff insufficiency	0.34	0.01	≈0.03 [CI not recoverable]

All values were derived by the authors from elective PTIRs and 95% CIs in Table 3.S13 [13], not reported by the NJR. PTIR ratios were computed on the log scale. The 95% CI for cuff insufficiency was unrecoverable because the rounded 95% CI for RTSR [0.00, 0.01] includes 0. Because indications are not mutually exclusive, and 15.3% of revisions had no recorded indication, PTIR ratios describe recorded indications, not adjudicated causes. All estimates are unadjusted.

## Data Availability

The data presented in this study are available in the 22nd Annual Report of the National Joint Registry (Section 3.S, Tables 3.S1–3.S21) at https://reports.njrcentre.org.uk and www.njrcentre.org.uk. No new patient-level data were created. The author-derived quantities in Table 3 and Table 4 and Figure 1, Figure 2, Figure 3 and Figure 4 are reproducible from the cited registry tables using the formulae in Section 2.4; the extraction sheet and Python scripts used for calculation and figure generation are available from the corresponding author on request.

## Supplementary Materials

The following supporting information can be downloaded at: https://www.mdpi.com/article/10.3390/jcm15218381/s1, Table S1: Reported indications for first revision (PTIR, 95% CI) after elective anatomic TSR, RTSR and hemiarthroplasty (NJR Table 3.S13); Table S2: Cumulative percentage revision (95% CI) by sex and age across all elective primary shoulder replacements (NJR Table 3.S7); Supplementary Note S1: Surgeon volume.
