# Supplementary material for: Timing and Reported Indications for Revision After Anatomic Versus Reverse Total Shoulder Replacement: A National Joint Registry Study
Source: J Clin Med. 2026 Oct 28;15(21):8381. doi: 10.3390/jcm15218381 (PMC13763872; doi:10.3390/jcm15218381)
Supplement: Supplementary file 1 [file jcm-15-08381-s001.zip › jcm-4616560-supplementary.pdf]

Supplementary Materials

Timing and Reported Indications for Revision After Anatomic Versus Reverse Total Shoulder Replacement: A National Joint Registry Study

Sedeek Mosaid, Banah Khoshnaw, Nuala Pepper, Omar Salem, Paul Lee and Charles Corbin

Reference numbers in this file refer to the reference list of the main text. All values are taken from, or derived from, the National Joint Registry (NJR) 22nd Annual Report 2025 [13]; the period covered is April 2012 to 31 December 2024 (elective primary shoulder replacements, England and Wales).

Table S1. Reported indications for first revision (PTIR, 95% CI) after elective anatomic TSR, RTSR and hemiarthroplasty (NJR Table 3.S13).

Construct	All-cause	Infection	Instability/ dislocation	Cuff insufficiency	Aseptic loosening/lysis	Periprosthetic fracture	Other
Anatomic TSR	0.72 [0.67, 0.77]	0.07 [0.05, 0.08]	0.19 [0.17, 0.22]	0.34 [0.31, 0.37]	0.16 [0.14, 0.18]	0.04 [0.03, 0.05]	0.20 [0.18, 0.23]
RTSR	0.64 [0.60, 0.68]	0.17 [0.15, 0.19]	0.24 [0.21, 0.26]	0.01 [0.00, 0.01]	0.11 [0.09, 0.12]	0.07 [0.06, 0.09]	0.15 [0.13, 0.17]
Hemiarthroplasty (context)	1.27 [1.19, 1.36]	0.08 [0.06, 0.11]	0.12 [0.09, 0.15]	0.44 [0.39, 0.50]	0.07 [0.06, 0.10]	0.05 [0.03, 0.07]	0.63 [0.57, 0.69]

Source: Data are taken from NJR Table 3.S13 [13]. Because indications are not mutually exclusive, component rates do not sum to the all-cause rate. Events/prosthesis years at risk: anatomic TSR 851/118,730; RTSR 1077/168,260; hemiarthroplasty 802/63,070 [13]. PTIR, prosthesis-time incidence rate; RTSR, reverse total shoulder replacement; TSR, total shoulder replacement; CI, confidence interval.

Table S2. Cumulative percentage revision (95% CI) by sex and age across all elective primary shoulder replacements (NJR Table 3.S7).

Sex	Age (years)	N	5 years	10 years	12 years
Female	<55	1765	8.91 [7.54, 10.52]	13.92 [11.84, 16.32]	19.07 [15.47, 23.38]
Female	≥75	25,864	2.26 [2.06, 2.47]	2.80 [2.55, 3.09]	2.96 [2.63, 3.33]
Male	<55	2525	10.64 [9.34, 12.11]	16.95 [14.91, 19.24]	23.52 [19.67, 27.98]
Male	≥75	7871	4.75 [4.24, 5.30]	6.22 [5.40, 7.15]	6.22 [5.40, 7.15]

Source: NJR Table 3.S7 (all elective primary shoulder replacements) [13]. Intermediate age bands (55–64 and 65–74 years) are omitted for brevity; the full data are available in the NJR Annual Report [13]. In the source table, the female and male 'All' subtotals (51,884 and 24,130) sum to 76,014, whereas the elective total reported in Tables 3.S2 and 3.S6 is 76,012; this two-case inconsistency is present in the source report, and totals should not be constructed from the sex subtotals. CI, confidence interval.

Supplementary Note S1. Surgeon volume

In 2024, elective and trauma shoulder replacements were performed by 552 consultants at a median of 14 procedures each (IQR 6.5–26). The median consultant volume increased from 4.5 in 2012 to 14 in 2024, apart from a decrease to 7 in 2020 (the COVID-19 pandemic), suggesting gradual consolidation [13]. Separately, the NJR/HES analysis by Valsamis et al. identified a threshold of 10.4 procedures per year, below which the risk of revision increased as much as twofold (hazard ratio 1.94, 95% CI [1.27, 2.97]) [12]. These two figures are not directly comparable – the NJR distribution pools elective and trauma

procedures in a single year, whereas the threshold concerns modelled mean annual elective volume – so we report them side by side without inferring the exact proportion of practice below the threshold.

Source: NJR Table 3.S3 [13] and Valsamis et al. [12]. HES, Hospital Episode Statistics; IQR, interquartile range.
